# Large Cutaneous Mixed Tumor of Apocrine Type Formerly Known As Chondroid Syringoma: A Case Report

**DOI:** 10.7759/cureus.109772

**Published:** 2026-05-27

**Authors:** Eva Bernstein, John J Fowler, Morteza Khodaee

**Affiliations:** 1 Family Medicine, University of Colorado School of Medicine, Denver, USA; 2 Pathology, University of Colorado School of Medicine, Aurora, USA; 3 Sports Medicine, University of Colorado School of Medicine, Denver, USA

**Keywords:** dermatology, excisional biopsy, pathology, point-of-care ultrasound, subcutaneous mass

## Abstract

Large subcutaneous masses are relatively uncommon and usually benign. Lipomatous tumors predominate and are typically nonmalignant; however, atypical lipomatous tumors and liposarcomas can occur. Although malignancy is uncommon overall, soft tissue sarcomas may present as large or deep subcutaneous masses. A woman in her 50s presented to the primary care clinic with a large, firm, and mobile mass on the posterior aspect of the left shoulder that had been present for seven years. After excisional biopsy, pathological evaluation confirmed the diagnosis of cutaneous mixed tumor, apocrine type (chondroid syringoma). Cutaneous mixed tumor is a rare and typically benign neoplasm that can arise from either eccrine or apocrine glands and typically presents as a slow-growing, painless nodule. Definitive management and treatment involve surgical excision with a goal of removal of the entire tumor to avoid both recurrence and rare instances of malignant transformation.

## Introduction

Subcutaneous masses are a frequent clinical finding, although their incidence is difficult to quantify and likely underestimated [1-3]. The majority are benign, most commonly lipomas, angiolipomas, epidermal cysts, and fibromas [1-3]. Lipomatous tumors are the most prevalent, with the majority being benign; however, atypical lipomatous tumors and liposarcomas, though rare, pose significant risk [2,4]. While the overall incidence of malignant subcutaneous tumors is low, a substantial proportion of soft tissue sarcomas and other malignancies may present as large or deep masses, and even lesions smaller than 5 cm can be malignant [1,4-6].

Clinical evaluation should focus on features suggestive of malignancy, including size larger than 5 cm, rapid growth, deep (subfascial) location, sudden onset, and imaging findings such as fascial involvement, lobulation, hemorrhage, necrosis, peritumoral edema, and skin thickening [1,2,4,7]. Notably, malignant lesions may be smaller than 5 cm, and thus size alone is insufficient for risk stratification [3-6]. Additional concerning features include abundant vascularity on ultrasound and atypical radiographic characteristics such as intramuscular location, septations, nonfat nodules, heterogeneity, and ill-defined margins [2,3].

Management strategies for large subcutaneous masses are guided by risk assessment. Benign lesions may be observed or excised if symptomatic or diagnostically uncertain [1,4,8-10]. For masses with high-risk features, MRI is the imaging modality of choice, supplemented by ultrasound or CT as indicated [1-7]. Biopsy is recommended for deep lesions larger than 5 cm or any mass with concerning clinical or radiographic features [2-4,8,10]. Definitive management of malignant tumors requires wide surgical excision with oncologically appropriate margins, often in conjunction with multidisciplinary input [2,4,8,10].

## Case presentation

**Figure 1:**
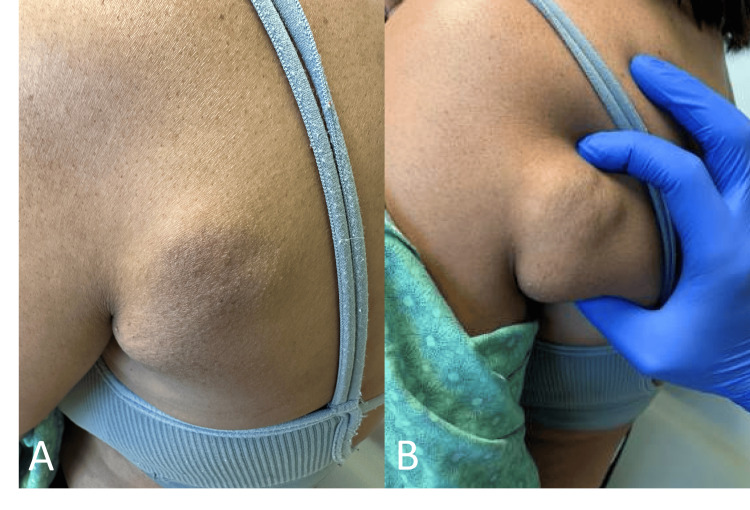
(A, B) A non-tender, mobile subcutaneous mass measuring approximately 7 × 5 × 5 cm located over the left posterior shoulder.

A woman in her 50s presented to the primary care clinic with a large, firm, and mobile mass on the posterior aspect of the left shoulder. She reported this mass had been present for seven years, initially associated with a spider bite and slowly growing over time, but stable in size for the past five years. She reported some pain with stretching movements and therefore wanted it removed. Physical examination revealed a firm, mobile, non-tender mass measuring approximately 5 × 7 cm that was adherent to the overlying skin (Figure 1 and Video 1). There was no palpable lymphadenopathy in the region.

**Video 1:** The mass was freely mobile and adherent to the overlying skin but not adherent to the underlying deep tissues, including the muscle fascia.

**Figure 2:**
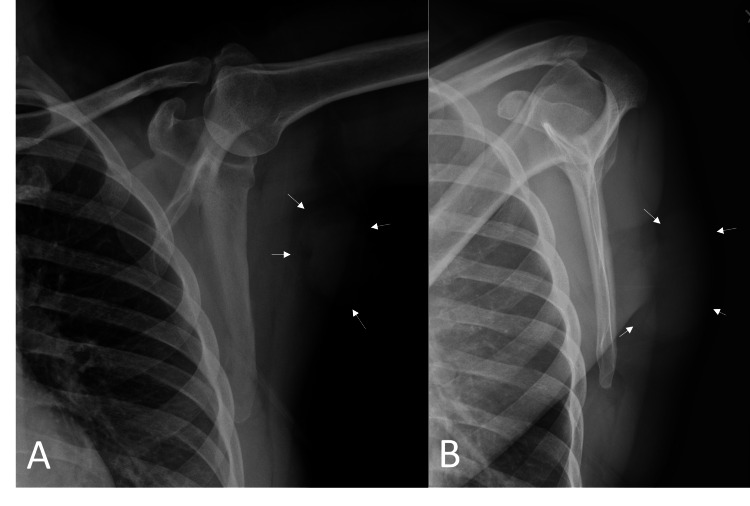
Plain radiography images (A, B) reveal a soft tissue mass (arrows) with no any bony abnormality.

Plain radiography showed a large (approximately 5 x 5 cm) nodular soft tissue density overlying the scapula (Figure 2).

**Figure 3:**
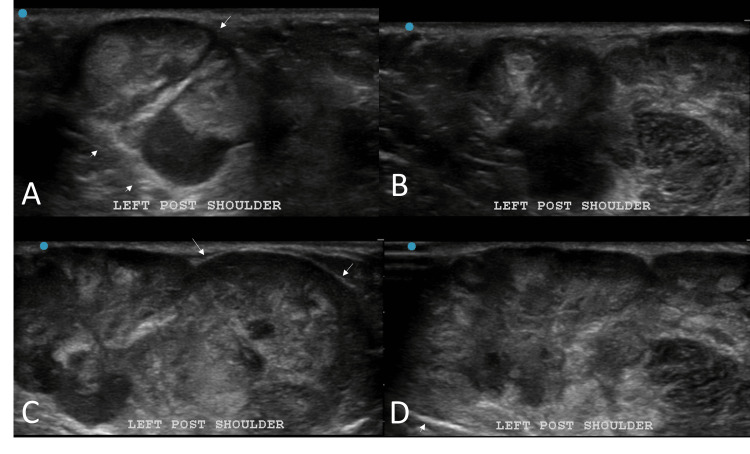
(A-D) Point-of-care ultrasound revealed a large (7.0 x 4.0 x 3.5 cm) encapsulated (arrows) subcutaneous mass with mixed hypoechoic and hyperechoic regions.

Point-of-care ultrasound showed a non-homogeneous, capsulated mass with areas of hyperechoic and hypoechoic regions (Figure 3).

**Figure 4:**
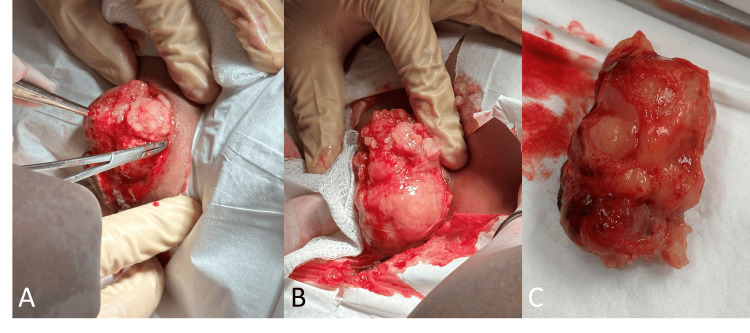
(A-C) The mass was completely excised through a marginal surgical incision.

At this stage, the differential diagnosis for a firm, large mobile soft tissue mass remained broad, encompassing benign lesions such as lipomas, fibromas, hamartomas, cysts, and neurofibromas, as well as malignant neoplasms such as soft tissue sarcomas. Masses measuring greater than 5 cm in diameter warrant further evaluation with advanced imaging or referral to a specialist, given the increased risk of malignancy. After discussing benefits and risks, she agreed on an excisional biopsy, which was performed in the office during the next visit (Figure 4). This was a marginal excision, as the tumor abutted the surgical margin but was not transected. Such a margin would be considered inadequate for the excision of a malignant tumor.

**Figure 5:**
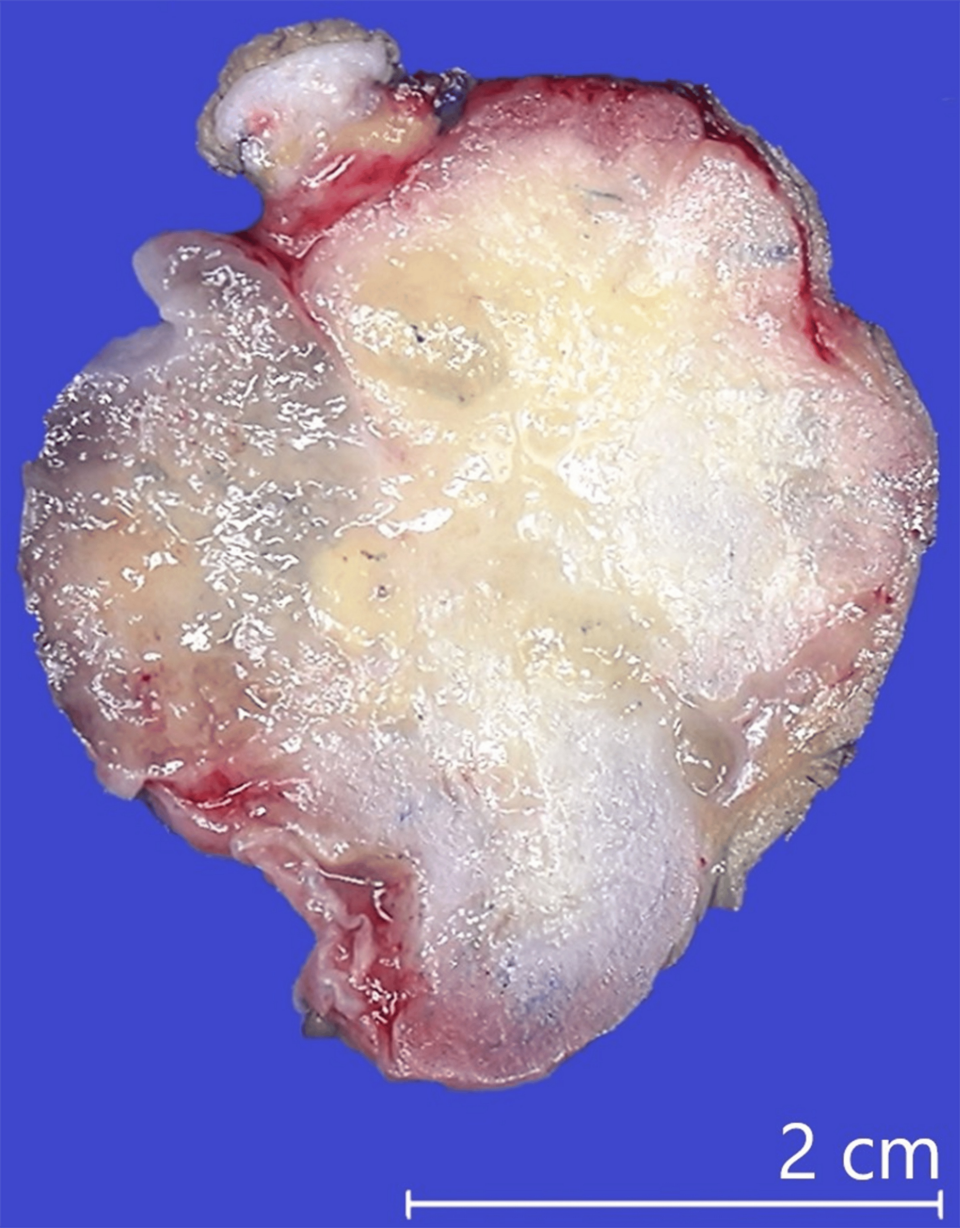
On gross examination, sections show a relatively well-circumscribed mass with a variably gelatinous to coarse grey-white cut surface.

**Figure 6:**
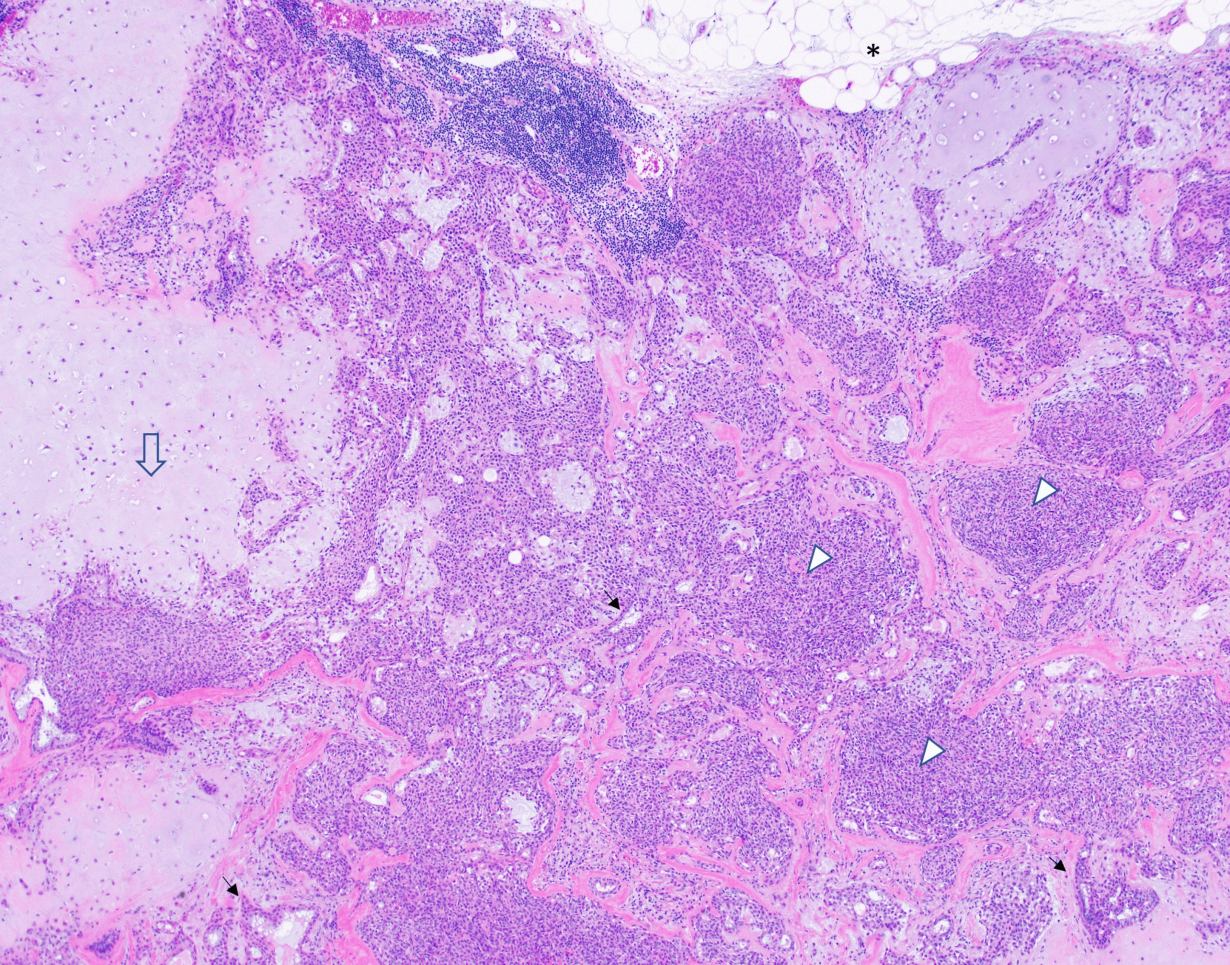
Sections show a large, circumscribed nodule comprised of epithelial, myoepithelial, and mesenchymal elements (H&E, 40 × magnification). The epithelial component (black arrows) is comprised of scattered tubules lined by columnar cells, while the myoepithelial component (white arrowheads) is comprised of expansive, syncytial nodules of polygonal cells. Lobules of mature cartilage comprise the mesenchymal component (open arrow). Mature adipose tissue is present at the periphery of the tumor (*).

**Figure 7:**
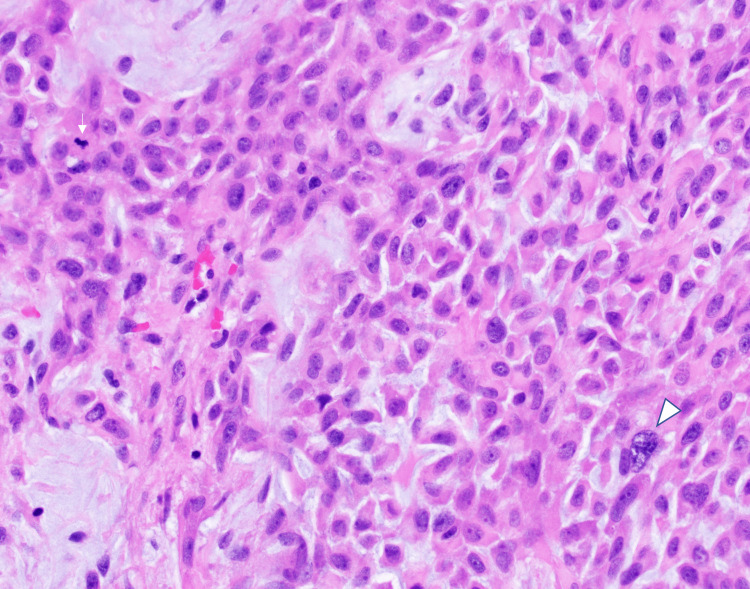
The prominent myoepithelial component is composed of sheets of polygonal and plasmacytoid cells with abundant eosinophilic cytoplasm (H&E, ×400 magnification). Rare mitotic-like figures are identified (white arrow), but proliferative activity by Ki-67 immunohistochemistry (not shown) was low. Random nuclear atypia is also present, with some myoepithelial cells demonstrating enlarged nuclei and irregularly distributed vesicular chromatin (white arrowhead). Mixed tumors with a prominent myoepithelial component may exhibit some cytologic atypia; however, this finding alone should not be interpreted as indicative of malignancy.

Gross evaluation showed a relatively well-circumscribed but unencapsulated 7.0 x 3.8 x 3.5 cm mass with a heterogeneous consistency. Some areas were soft, pale yellow, and gelatinous, whereas others were firm with a coarse cut surface and pale grey- to white cut surface (Figure 5). No firm, fibrotic areas around the periphery of the tumor, to suggest an infiltrative margin, were identified; nor were there foci of hemorrhage or friable consistency to suggest necrosis. On histopathologic examination, the tumor was comprised of nodules of mature cartilage and dense collagenous stroma punctated by well-formed branching tubules lined with a columnar epithelium, with a prominent associated population of variably plasmacytoid to cuboidal epithelial cells (Figures 6-7). These latter cells formed syncytial nodules. Rare mitotic-like figures were identified (Figure 7), but even in these areas, proliferative activity, as assessed by Ki67 immunohistochemistry, was low (immunopositivity in fewer than 1% of these cells). There was focal atypia characterized by nuclear enlargement and irregularly distributed chromatin (Figure 7). Such cells were distributed throughout the lesion. There was no anaplasia, atypical mitotic activity, necrosis, or infiltration beyond the circumscribed margins of the tumor. Neither normal breast nor salivary gland parenchyma was identified in association with the lesion. These findings were taken to establish the diagnosis of cutaneous mixed tumor, apocrine type (Figures 6-7).

**Figure 8:**
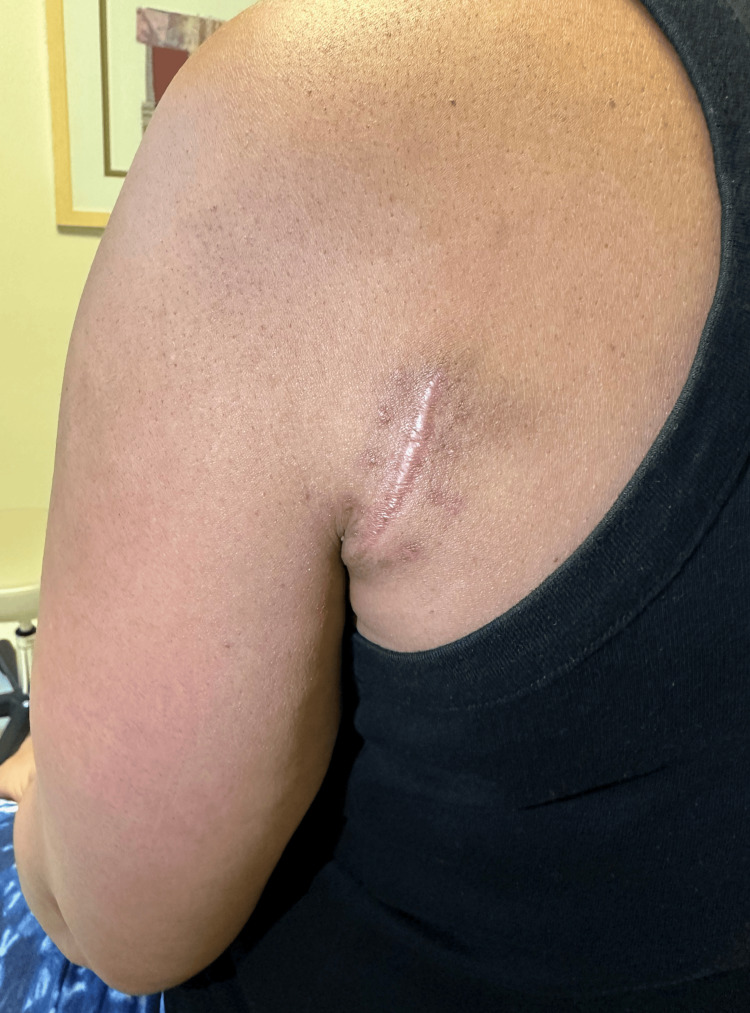
Healed surgical scar at the site of excision, four months postoperatively.

At the follow-up visit four months postoperatively, the surgical wound demonstrated appropriate healing (Figure 8).

## Discussion

Cutaneous mixed tumor (formerly chondroid syringoma) is a rare and typically benign neoplasm that accounts for less than 0.1% of all primary skin tumors [11,12]. It can arise from either eccrine or apocrine glands and typically presents as a slow-growing, painless nodule [11-15]. It is widely recognized as the skin equivalent of a pleomorphic adenoma. The cause of these tumors is unclear, but some studies have suggested an association with trauma or chronic irritation [11-17].

The diagnosis of cutaneous mixed tumor is challenging due to its nonspecific clinical appearance, which can mimic epidermoid cysts, dermoid cysts, neurofibromas, basal cell carcinomas, and metastatic lesions, particularly when arising in atypical locations such as the axilla [11-13]. It most frequently presents as a slow-growing, painless, firm, and well-circumscribed nodule; however, lesions can be solitary or multiple, and overlying skin can be normal or erythematous with crusting features [11-16]. Considering the wide variety of presentations, definitive diagnosis requires excision or biopsy for histopathological examination [11-19].

Generally, the striking and unique histopathologic appearance of cutaneous mixed tumor affords a relatively straightforward diagnosis. The presence of banal mesenchymal components, such as cartilage, bone, adipocytes, or prominent fibrosis, admixed with epithelial cells forming branched tubules (in the apocrine type), and myoepithelial cells [11-19]. These lesions show substantial histomorphologic overlap with pleomorphic adenoma of the salivary gland and fibroadenoma of the breast [14,16-18]. Correlation with clinical findings and imaging characteristics, therefore, is of utmost importance in excluding these, particularly in lesions that arise on the cheek or lip, orbit, or external auditory meatus, and lesions that arise in or around skin covering the breast, axilla, or anogenital region [11,13,16-18].

Exclusion of malignant transformation by histopathologic analysis is essential. Diagnosis of malignant mixed tumor requires the presence of atypical tumor cells arranged as sheets, glands, or papillary structures (adenocarcinoma), marked pleomorphism, and atypical mitotic activity (myoepithelial carcinoma), or frank sarcomatoid transformation (sarcomatoid carcinoma) [11-19]. However, care must be taken when considering the classification of a myoepithelial component as malignant. A prominent myoepithelial component may exhibit some cytologic atypia, but this finding alone should not be taken to indicate malignancy [17,18].

Definitive treatment of cutaneous mixed tumors consists of complete surgical excision to minimize the risk of local recurrence and the rare possibility of malignant transformation [1,4,11-20]. Surgical excision may be classified according to the margin achieved, including intralesional, marginal, wide, and radical resection, as well as excisional biopsy [20]. Intralesional resection leaves gross or microscopic residual tumor and includes procedures such as debulking, curettage, or tumor transection at the surgical margin. In contrast, marginal resection removes the tumor along its pseudocapsule with only a minimal amount of surrounding tissue. Although no gross tumor remains, microscopic residual disease may be present; if the tumor is identified at the margin, the procedure is more appropriately classified as intralesional [20]. In some cases, an excisional biopsy is effectively equivalent to a marginal resection.

In the present case, the lesion was removed by marginal resection. Given the benign histopathology, either close clinical surveillance or re-excision to obtain a small cuff of uninvolved tissue may be considered. However, had there been definite tumor involvement at the surgical margin, the procedure would have been classified as an intralesional resection, and re-excision would have been recommended because of the increased risk of local recurrence associated with incomplete excision.

Long-term follow-up is advisable, particularly for large lesions or those with atypical clinical or histologic features. Awareness of cutaneous mixed tumors and their inclusion in the differential diagnosis of cutaneous and subcutaneous masses is important to facilitate accurate diagnosis and appropriate management.

## Conclusions

Evaluation of large subcutaneous masses should focus on features suggestive of malignancy, including size greater than 5 cm, rapid growth, deep (subfascial) location, sudden onset, and concerning imaging findings such as fascial involvement, lobulation, hemorrhage, necrosis, peritumoral edema, or skin thickening. These lesions are relatively uncommon, and no universally accepted clinical algorithm exists to guide their evaluation. Careful clinical monitoring is essential, with further investigation warranted when concerning features are present, particularly rapid growth, pain, or tenderness. In such cases, additional diagnostic evaluation, including imaging and biopsy, should be considered. Point-of-care ultrasound is a valuable adjunct for identifying abscesses, characterizing encapsulated lesions, and guiding procedures. Histopathologic examination remains the gold standard for diagnosis and is essential to exclude malignancy. Cutaneous mixed tumor is a rare, typically benign neoplasm of eccrine or apocrine origin that usually presents as a slow-growing, painless nodule. Definitive management consists of complete surgical excision to minimize recurrence and prevent rare malignant transformation.
